# Increased flow limitation during sleep is associated with decreased psychomotor vigilance task performance in individuals with suspected obstructive sleep apnea: a multi-cohort study

**DOI:** 10.1093/sleep/zsae077

**Published:** 2024-03-21

**Authors:** Eric Staykov, Dwayne L Mann, Timo Leppänen, Juha Töyräs, Samu Kainulainen, Ali Azarbarzin, Brett Duce, Scott A Sands, Philip I Terrill, The STAGES Cohort Investigator Group

**Affiliations:** School of Electrical Engineering and Computer Science, University of Queensland, Brisbane, QLD, Australia; School of Electrical Engineering and Computer Science, University of Queensland, Brisbane, QLD, Australia; School of Electrical Engineering and Computer Science, University of Queensland, Brisbane, QLD, Australia; Department of Technical Physics, University of Eastern Finland, Kuopio, Finland; Diagnostic Imaging Center, Kuopio University Hospital, Kuopio, Finland; School of Electrical Engineering and Computer Science, University of Queensland, Brisbane, QLD, Australia; Department of Technical Physics, University of Eastern Finland, Kuopio, Finland; Science Service Center, Kuopio University Hospital, Kuopio, Finland; Department of Technical Physics, University of Eastern Finland, Kuopio, Finland; Diagnostic Imaging Center, Kuopio University Hospital, Kuopio, Finland; Division of Sleep and Circadian Disorders, Department of Medicine, Brigham & Women’s Hospital & Harvard Medical School, Boston, MA, USA; Department of Respiratory and Sleep Medicine, Princess Alexandra Hospital, Brisbane, QLD, Australia; Institute of Health and Biomedical Innovation, Queensland University of Technology, Brisbane, QLD, Australia; Division of Sleep and Circadian Disorders, Department of Medicine, Brigham & Women’s Hospital & Harvard Medical School, Boston, MA, USA; School of Electrical Engineering and Computer Science, University of Queensland, Brisbane, QLD, Australia

**Keywords:** flow limitation, vigilance, response speed, sleepiness, ventilatory burden, apnea–hypopnea index, hypoxic burden, polysomnography, obstructive sleep apnea, respiratory events

Approximately one billion adults worldwide between the ages of 30 and 69 years have obstructive sleep apnea (OSA) [1]. Despite substantial evidence that demonstrates patients with OSA have impaired vigilance, the standard clinical measurement of OSA severity (the apnea–hypopnea index [AHI]) is not clearly associated with psychomotor vigilance task (PVT) performance, which is an objective measurement of vigilance [2]. One reason for this is that while the AHI quantifies the frequency of respiratory events, it does not quantify the extent to which ventilation fails to meet intended levels, i.e. the severity of airway flow limitation.

We recently quantified the association between overnight flow limitation and vigilance using data from 998 individuals with suspected OSA who undertook both PVT and diagnostic polysomnography at the Princess Alexandra Hospital in Brisbane, Australia (“PA cohort”) [3]. We found that the burden of flow limitation during sleep was associated with vigilance impairment, independent of the AHI and other domains of OSA pathology such as arousal severity and hypoxemia severity. Here we extended this investigation using an independent cohort of 324 participants from the Stanford Technology Analytics and Genomics in Sleep (STAGES) study and incorporated ventilatory burden [4, 5] into the analysis. We hypothesized that the association between overnight flow limitation severity and vigilance would be consistent between cohorts.

## Materials and Methods

STAGES is a cross-sectional, multi-center study conducted between 2018 and 2020 to improve detection, treatment, and prevention of sleep disorders, and better understand the genetics of sleep. Diagnostic polysomnograms were available on The National Sleep Research Resource [6]; analysis was approved by The University of Queensland Research Ethics and Integrity unit (2023/HE000064). Of the 1687 studies available, 488 were recorded at the Stanford Sleep Medicine Center (Redwood City, United States) and contained unfiltered nasal pressure airflow needed for analysis. In addition to previously described exclusion reasons [3], participants with < 2 hours of total analyzable sleep time or a primary suspicion other than sleep-related breathing disorder were excluded, resulting in 324 analyzed participants (Supplementary Figure S1).

Participants completed an in-laboratory 3-minute PVT on the evening of the polysomnography. The primary outcome was mean response speed (also known as reciprocal response time and herein referred to as “PVT speed”). This metric is comparable between 3- and 10-minute PVT versions [7], and was calculated by reciprocally transforming each response time, then averaging the values. One participant with two consecutive response times exceeding 30 seconds was excluded because of suspected protocol nonadherence.

Flow limitation severity during sleep was quantified on a breath-by-breath basis using the nasal pressure airflow signal (DC amplified, 32 Hz sampling rate) [8]. A published method [9] was used to correct for reduced airflow signal-to-noise ratio. Hypoxic burden and two measures of ventilatory burden were quantified as covariates [4, 5].

**Figure 1.**
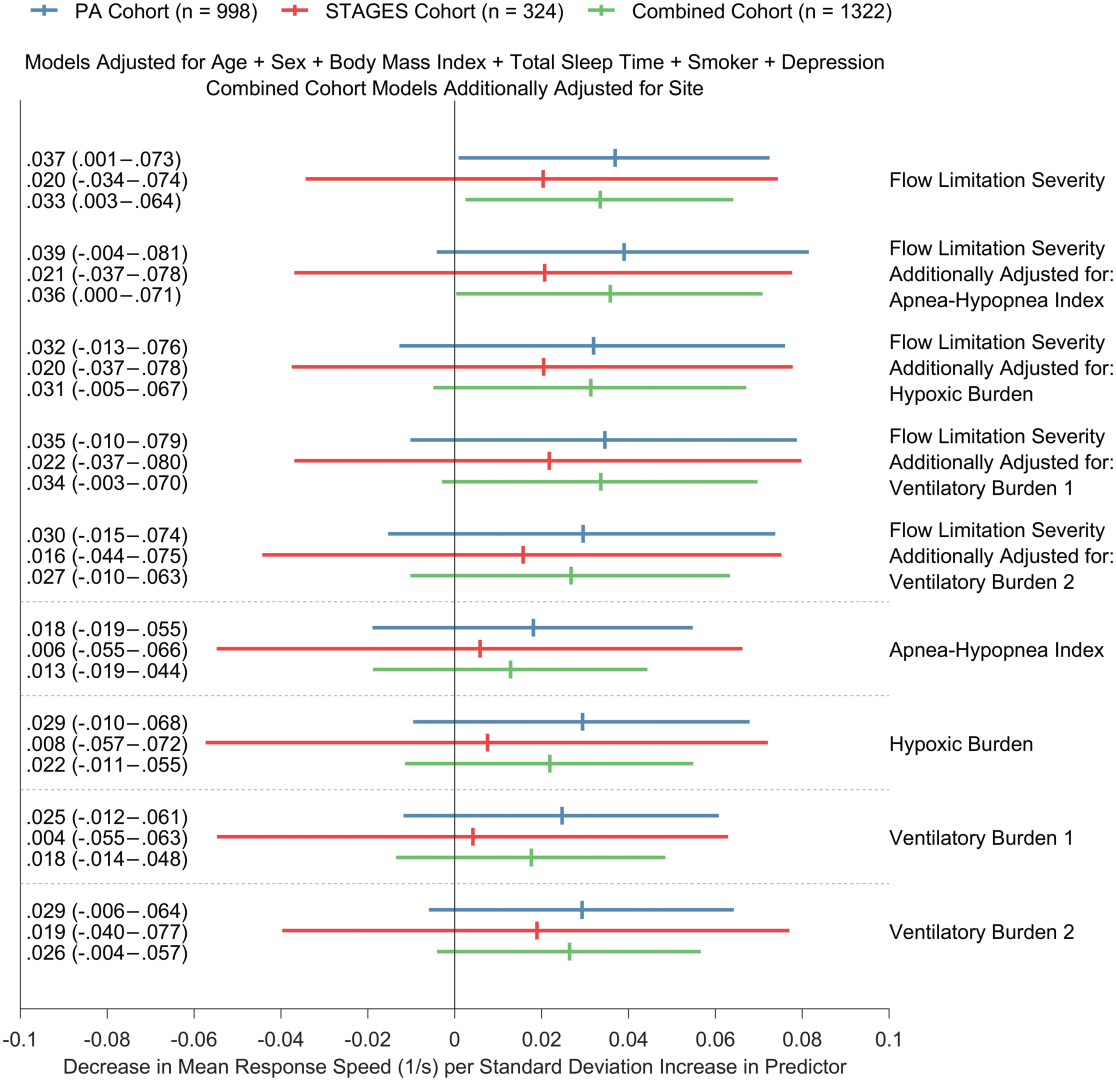
Association between one standard deviation increase in predictor variables and decreased psychomotor vigilance task mean response speed. Vertical ticks represent coefficient estimates and horizontal bars represent 95% confidence intervals. The combined cohort (*n* = 1322) was composed of *n* = 324 participants from the STAGES cohort and *n* = 998 participants from the Princess Alexandra Hospital cohort. Multivariable linear regression models were adjusted for sex, smoking status, depression, total sleep time, body mass index, and age. “Site” was added as a binary categorical term in the combined cohort analyses to compensate for differences between cohort characteristics not captured by standard covariates. Box-Cox transformation values derived in the combined cohort were used to back-transform mean response speed into comparable units. Ventilatory burden 1 was computed as described in [4], with minor modifications. Ventilatory burden 2 is the percentage of breaths during sleep under 50% eupnea, inspired by Parekh et al. [5].

The association between flow limitation severity and PVT speed was assessed using multivariable linear regression adjusting for covariates (see Figure 1 caption). Initial analysis was performed in the STAGES and Princess Alexandra (PA) cohorts alone, followed by an analysis of the combined cohort (STAGES and PA). Data were first transformed and standardized in respective cohorts, then combined to adjust for differences in PVT versions and other cohort characteristics. “Site” was also added as a binary categorical term in the combined cohort analyses to compensate for differences between cohorts not captured by standard covariates. Significance level threshold was *p* < 0.05. Refer to the Supplementary Methods for more information.

## Results

Compared to the PA cohort, participants in the STAGES cohort were younger, had more total sleep time, faster PVT speed, and lower body mass index, prevalence of smoking, flow limitation severity, AHI, hypoxic burden, and ventilatory burden (all *p* < 0.001, Supplementary Table S1). There were no differences in sex distribution or prevalence of depression. Flow limitation severity was no more than moderately correlated with the AHI, hypoxic burden, and two measures of ventilatory burden (Pearson’s *r* ≤ 0.66, Supplementary Figure S2).

In models that assessed the overall severity of flow limitation during sleep and PVT speed, effect sizes were similar between the PA (0.037 1/s) and STAGES (0.020 1/s) cohorts (Figure 1). Flow limitation severity was not significantly associated with PVT speed in the STAGES cohort (*p* = 0.466). In the combined cohort, one standard deviation (16.4%) increase in flow limitation severity was associated with a 0.033 1/s decrease in PVT speed (95% CI: 0.003 to 0.064, *p* = 0.034). Flow limitation severity remained a statistically significant predictor when the AHI was added as a covariate (*p* = 0.048), but not when hypoxic burden or ventilatory burden were added as covariates (*p* ≥ 0.071). The AHI, hypoxic burden, and ventilatory burden were not significantly associated with PVT speed (*p* ≥ 0.089).

## Discussion

The objective of this study was to extend our findings from the PA cohort using the STAGES cohort. We hypothesized that the association between overnight flow limitation severity and vigilance in participants with suspected OSA would be consistent between cohorts. Albeit not significant within 95% confidence in the STAGES cohort, the magnitude of association between flow limitation and PVT speed was similar to the value reported in the substantially larger PA cohort (Supplementary Table S3), suggesting consistency of this relationship. Notably, analysis of the combined cohort (1322 participants) confirmed that increased flow limitation severity during sleep is significantly associated with decreased PVT speed (with smaller confidence interval than in the PA cohort alone). Exploratory analyses of other PVT measures revealed similar associations (Supplementary Table S4). Flow limitation was associated with PVT speed independent of Epworth Sleepiness Scale score, sex, and age (Supplementary Table S5). The AHI was not associated with PVT performance, consistent with other studies [2, 3].

Three mechanisms can explain the association between nocturnal flow limitation and daytime hypovigilance in OSA. First, EEG microstructure during sleep might be modified due to flow limitation, resulting in daytime hypovigilance [10]. Second, flow limitation may cause hypercapnia and acute respiratory acidosis leading to cognitive impairment that persists throughout the day [3, 11]. Third, flow limitation may cause increased work of breathing leading to daytime fatigue [2]. Further studies of these intermediate mechanisms are now warranted.

Hypoxic burden and ventilatory burden have previously been shown to be associated with cardiovascular disease and all-cause mortality [4, 5]. In this study, we did not detect a significant association between hypoxic burden and ventilatory burden and PVT speed, likely due to low prevalence of severe OSA in the STAGES cohort. Therefore, directly estimating the upstream pharyngeal disturbance (flow limitation) may be more insightful with respect to vigilance risks than quantifying downstream sequelae such as lost ventilation and hypoxemia.

This study has several limitations. First, we did not find a significant association between flow limitation severity and PVT speed within the STAGES cohort alone. This can be explained by smaller sample size (324 participants) than the PA cohort. Supporting this explanation, a sensitivity analysis was conducted on 1000 random subsets of 324 participants from the larger PA cohort; no significant association was found (median *p* = 0.144) in these simulated smaller cohorts. Second, different PVT versions were used between cohorts. Mean response speed was chosen as the primary outcome because it is one of the most comparable metrics between 3-minute and 10-minute PVT versions [7]. Furthermore, data were transformed and standardized in respective cohorts, then combined to adjust for differences in PVT versions and other cohort characteristics. Finally, measures of arousal severity [3] were not explored because arousal scoring was unavailable in the STAGES cohort.

Overall, increased nocturnal flow limitation severity was associated with decreased vigilance using two cohorts of individuals with suspected OSA. Patients who are likely to have improved vigilance with OSA treatment could be identified by using flow limitation alongside patient symptoms and the AHI.

## Supplementary Material

zsae077_suppl_Supplementary_Figures_S1-S2_Tables_S1-S5

## Data Availability

The data from the Stanford Technology Analytics and Genomics in Sleep (STAGES) study is available from the National Sleep Research Resource website. All other data and code will be shared on request to the corresponding author.

## Clinical Trial

This study involved retrospective analysis of diagnostic polysomnograms and therefore does not qualify as a clinical trial.
