# Supplementary material for: Increased flow limitation during sleep is associated with decreased psychomotor vigilance task performance in individuals with suspected obstructive sleep apnea: a multi-cohort study
Source: Sleep. 2024 Mar 21;47(6):zsae077. doi: 10.1093/sleep/zsae077 (PMC11168759; doi:10.1093/sleep/zsae077)
Supplement: zsae077_suppl_Supplementary_Figures_S1-S2_Tables_S1-S5 [file zsae077_suppl_supplementary_figures_s1-s2_tables_s1-s5.docx]

# SUPPLEMENTARY MATERIAL

**Title:** Increased flow limitation during sleep is associated with decreased psychomotor vigilance task performance in individuals with suspected obstructive sleep apnea: a multi-cohort study

**Authors:** Eric Staykov^1,^*, Dwayne L. Mann^1^, Timo Leppänen^1,4,5^, Juha Töyräs^1,4,6^, Samu Kainulainen^4,5^, Ali Azarbarzin^7^, Brett Duce^2,3^, Scott A. Sands^7,#^, Philip I. Terrill^1,#^ and The STAGES Cohort Investigator Group^

**Affiliations**

^1^ School of Electrical Engineering and Computer Science, The University of Queensland, Brisbane, Australia.

^2^ Department of Respiratory & Sleep Medicine, Princess Alexandra Hospital, Brisbane, Australia.

^3^ Institute of Health and Biomedical Innovation, Queensland University of Technology, Brisbane, Australia.

^4^ Department of Technical Physics, University of Eastern Finland, Kuopio, Finland.

^5^ Diagnostic Imaging Center, Kuopio University Hospital, Kuopio, Finland.

^6^ Science Service Center, Kuopio University Hospital, Kuopio, Finland.

^7^ Division of Sleep and Circadian Disorders, Department of Medicine, Brigham & Women's Hospital & Harvard Medical School, Boston, MA, USA.

*Correspondence: Eric Staykov, School of Electrical Engineering and Computer Science, The University of Queensland, Brisbane, Australia. E-mail: e.staykov@uq.edu.au

^#^These authors contributed equally.

^This research has been conducted using the STAGES (Stanford Technology, Analytics and Genomics in Sleep) Resource funded by the Klarman Family Foundation. The investigators of the STAGES study contributed to the design and implementation of the STAGES cohort and/or provided data and/or collected biospecimens, but did not necessarily participate in the analysis or writing of this report. The full list of STAGES investigators can be found at the project website (https://sleepdata.org/datasets/stages).

# Supplementary Methods

## Polysomnographic recordings

Split-night studies that were included in the analysis had at least 2 hours of total analyzable sleep time before the onset of continuous positive airway pressure (CPAP) titration. All data during the CPAP titration period was excluded.

## Psychomotor vigilance task (PVT)

In the STAGES study, the Penn Computerized Neurocognitive Battery [1] was performed on the evening of the polysomnography. As part of the battery, vigilance was assessed using the Penn Psychomotor Vigilance Test. The test was administered in a proctored setting at the clinic using a laptop or desktop computer [2,3]. Participants were presented with a variable number of stimuli within 3 minutes in the STAGES cohort, whereas participants in the Princess Alexandra (PA) Hospital cohort performed a modified 10-minute PVT, i.e. 121 stimuli with no upper test time limit. For both PVT versions, there was no maximum time limit for each trial. However, response times were capped at 30 seconds in post hoc analyses as previously described [4]. More information specific to the PVT administered in the PA cohort has been published [5].

Mean response speed (also known as reciprocal response time) was chosen as the primary outcome metric based on the study conducted by Basner, Mollicone and Dinges [6], where participants took both the 10-minute and 3-minute PVT versions. It was demonstrated that mean response speed is one of the most comparable metrics between the two PVT versions. This was determined by examining the overlap in values, effect size magnitude, and percent reduction in effect size from 10-minute to 3-minute PVT versions. Lapses were defined as response times ≥ 355 ms for the 3-minute PVT and ≥ 500 ms for the 10-minute PVT [6].

## Quantification of flow limitation

We used a validated algorithm [7] to objectively assess the degree of flow limitation on a breath-by-breath basis using the nasal airflow signal. The gold standard used to train the algorithm was the ratio between pneumotach measured airflow and intra-esophageal diaphragm electromyography. This algorithm is based on multiple flow shape characteristics (e.g., fluttering and scooping) that describe non-rounded inspiratory/expiratory flow.

The primary exposure variable was flow limitation severity, which quantifies the mismatch between measured ventilation and intended ventilation (ventilatory drive). Specifically, flow limitation severity = (1 – (ventilation ÷ drive)) × 100. A score of 50% indicates ventilation was half of the intended level, and a score of 0% indicates a perfectly patent airway. The median value of flow limitation severity provided a single summary metric. All breaths during sleep including those that occurred within scored respiratory events were included in analyses. Breaths within obstructive apneas − scored apneas plus any unscored periods of ventilation below 10% of the local mean value (7-minute sliding window) − were imputed at the median respiratory rate and attributed a flow limitation severity of 99.9%.

Arousal scoring was not available in the STAGES cohort polysomnograms, so flow limitation severity during all sleep epochs including arousal breaths was used as the primary exposure variable in both cohorts. In the PA cohort, there was a very strong correlation between flow limitation severity including arousal breaths and flow limitation severity excluding arousal breaths (Pearson’s *r* = 0.996).

Estimation of nasal pressure airflow signal-to-noise ratio (SNR) involved splitting the airflow signal into 7-minute windows [8]. A published method [8] was used to correct for reduced airflow signal-to-noise ratio in the STAGES cohort. This correction factor had negligible effect in the PA cohort due to high airflow SNR (Supplementary Table S1).

Clipped breaths were automatically detected and excluded. Across all analyzed participants, an average of 0.07% of breaths were clipped, and the highest number of clipped breaths for one individual was 5.94% of total breaths.

## Other quantifications of OSA severity

Apneas and hypopneas were scored according to the American Academy of Sleep Medicine guidelines. The apnea-hypopnea index (AHI) was defined as the average number of apneas and hypopneas per hour during sleep. Arousal and desaturation scoring was not available in the STAGES cohort, so arousal severity and desaturation severity could not be quantified as we did in our previous study [5]. Hypoxic burden [9] was computed instead of desaturation severity [10] because hypoxic burden uses scored respiratory events to find resulting desaturations [9]. In the PA cohort, there was a very strong correlation between hypoxic burden and desaturation severity metrics (Pearson’s *r* = 0.953). Ventilatory burden 1 was computed as described [11], with two minor modifications: (1) average depth under 90% eupnea for each respiratory event was averaged instead of finding the average depth under 90% of the ensemble averaged ventilation signal, and (2) average event duration was calculated by averaging the duration of ventilation under 90% eupnea for each event instead of averaging manually scored event duration. Ventilatory burden 2 is the percentage of breaths during sleep under 50% eupnea, inspired by Parekh *et al*. [12].

## Statistical analyses

Data processing and statistical analyses were performed in R (version 4.2.2, R Foundation, Vienna, Austria) and MATLAB (version R2023b, Mathworks, Natick, MA, USA). All continuous variables were transformed to be normally distributed using the two-parameter Box-Cox [13] transformation $\text{Y' = }{\text{(Y+}\text{λ}_{\text{2}}\text{)}}^{\text{λ}_{\text{1}}}\text{-1) ÷ }\text{λ}_{\text{1}}$ or $\text{Y' =}\text{ }\text{log}_{\text{e}} \text{(Y+}\text{λ}_{\text{2}}\text{)}$ if $\text{λ}_{1}\text{ = 0}$. Optimal values of $\text{λ}_{\text{1}}$ and $\text{λ}_{\text{2}}$ were estimated using the boxcoxfit function from the geoR package (version 1.9-2) of R [14,15] and are presented in Supplementary Table S2. Variables were then standardized between 0 and 1. Box-Cox transformation parameters were derived using PA participants only or STAGES participants only for separate cohort analyses, and PA and STAGES cohorts combined for combined cohort analyses.

Initial linear regression analysis was performed in the PA and STAGES cohorts alone, followed by an analysis of the combined cohort. In the combined cohort analyses, data was first transformed and standardized in respective cohorts, then combined to adjust for differences in PVT versions and other cohort characteristics. “Site” was also added as a binary categorical term to compensate for differences between cohorts not captured by standard covariates. Multivariable linear regression models were developed to quantify the relationship between predictor variables and PVT and Epworth Sleepiness Scale (ESS) outcome variables. Models were adjusted for age, sex, and body mass index, total sleep time, smoking status, and depression. Significance level threshold was set at *p* < 0.05. Plots of model residuals were visually inspected for normality, zero mean, and constant variance. One participant from the STAGES cohort did not have ESS data and was excluded from analyses involving ESS.

# Supplementary Figures

## Figure S1


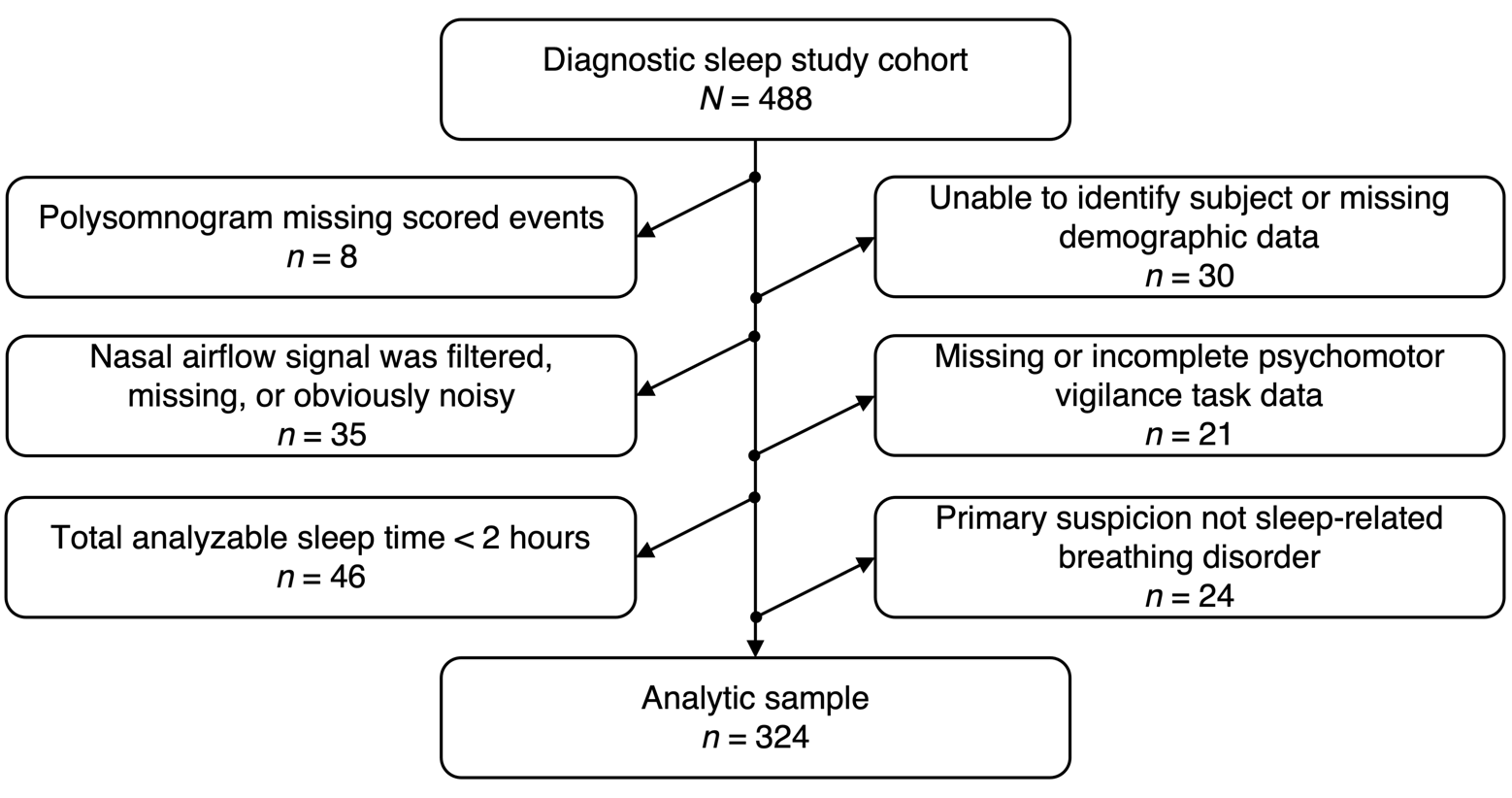


**Figure S1.** Flow diagram of participant exclusion criteria applied to the diagnostic sleep studies performed at the Stanford Sleep Medicine Center (Redwood City, United States). *n* = sample size.

## Figure S2


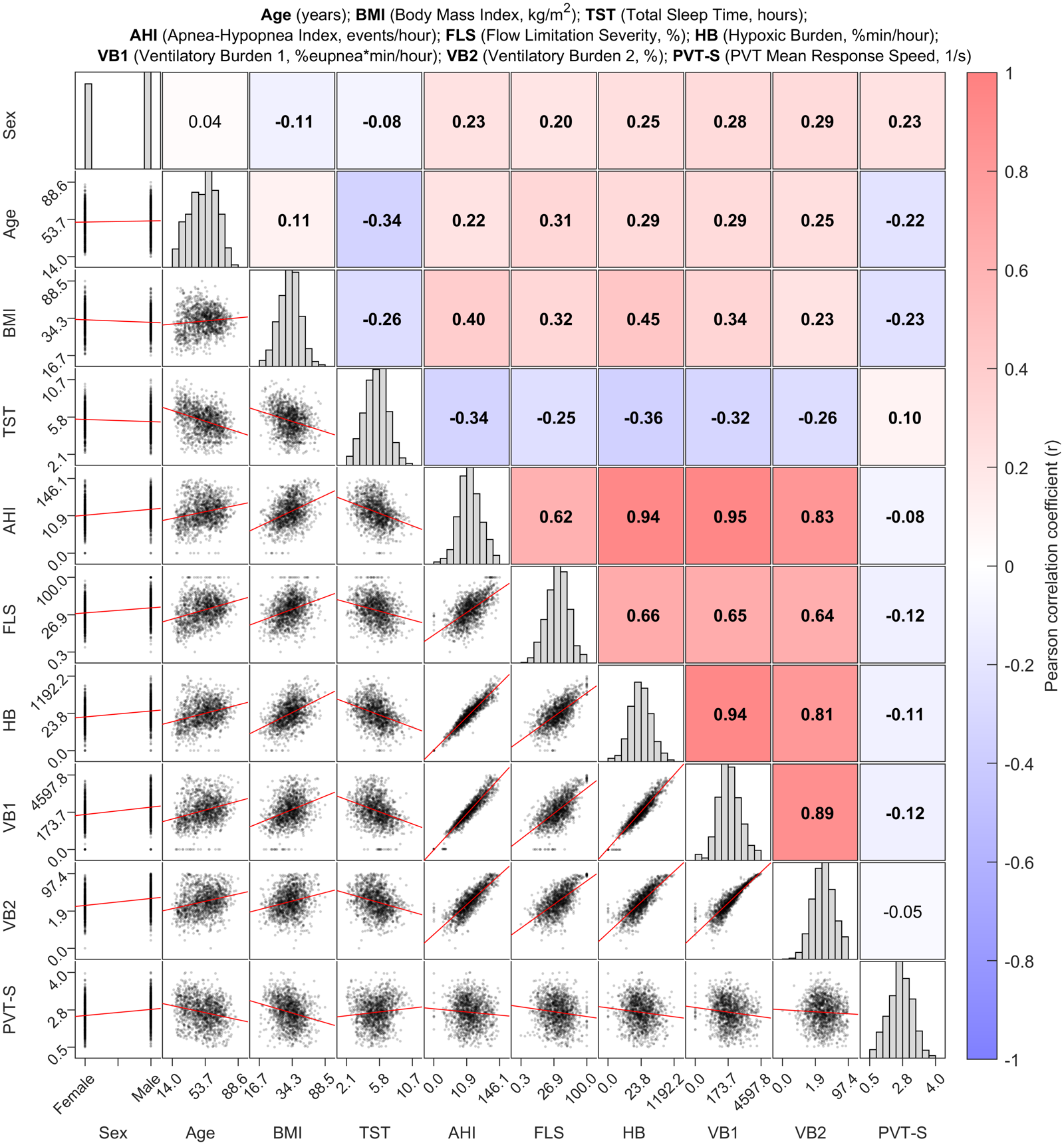


**Figure S2.** Histograms and scatter plots of demographic, predictor, and outcome variables (transformed and standardized, with back-transformed tick values). The combined cohort (*n* = 1322) was composed of *n* = 324 participants from the STAGES cohort and *n* = 998 participants from the PA cohort. Histograms along the diagonal show the distribution of each variable. Scatter plot regression lines are shown in red. Squares are colored and numbered based on Pearson's correlation coefficients (numbers in boldface represent *p* < 0.05). Axes scales are non-linear due to transformed data being plotted with back-transformed tick marks. Box-Cox transformation values derived in the combined cohort were used to back-transform variables.

# Supplementary Tables

## Table S1

**Table S1.** Comparison of demographic, polysomnographic, and psychomotor vigilance task (PVT) characteristics of the combined, STAGES, and Princess Alexandra (PA) Hospital cohorts.

|  | Combined Cohort | STAGES Cohort | PA  Cohort |
| --- | --- | --- | --- |
| Participants *n* (% male) | 1322 (53.2%) | 324 (54.0%) | 998 (52.9%) |
| Age (years) | 51.7 ± 14.9 | 42.0 ± 17.4 | 54.4 ± 13.5^a^ |
| Body Mass Index (kg/m^2^) | 32.2 ± 10.0 | 27.0 ± 7.8 | 34.0 ± 9.8^a^ |
| Smoker | 160 (12.1%) | 9 (2.8%) | 151 (15.1%)^b^ |
| Depression | 260 (19.7%) | 56 (17.3%) | 204 (20.4%) |
| Hypertension | 456 (34.5%) | 62 (19.1%) | 394 (39.5%)^b^ |
| Chronic Obstructive Pulmonary Disease | 103 (7.8%) | 10 (3.1%) | 93 (9.3%)^b^ |
| Type 2 Diabetes | 234 (17.7%) | 20 (6.2%) | 214 (21.4%)^b^ |
| Heart Failure | 48 (3.6%) | 10 (3.1%) | 38 (3.8%) |
| Stroke | 39 (3.0%) | 5 (1.5%) | 34 (3.4%) |
| Total Sleep Time (hours) | 5.4 ± 1.5 | 6.0 ± 2.0 | 5.2 ± 1.1^a^ |
| Non-Rapid Eye Movement Sleep (%) | 81.3 ± 11.3 | 78.7 ± 14.9 | 82.7 ± 7.6^a^ |
| Apnea-Hypopnea Index (events/hour) | 15.4 ± 26.5 | 10.5 ± 17.1 | 17.5 ± 29.0^a^ |
| Airflow Signal-to-Noise Ratio (dB) | 38.8 ± 8.3 | 31.3 ± 3.7 | 42.0 ± 5.4^a^ |
| Flow Limitation Severity (%) | 31.6 ± 16.4 | 27.2 ± 14.3 | 33.1 ± 16.9^a^ |
| Hypoxic Burden (%min/hour)^c^ | 22.5 ± 75.6 | 12.9 ± 33.3 | 27.5 ± 90.2^a^ |
| Ventilatory Burden 1 (%eupnea*min/hour) | 210.1 ± 577.3 | 99.9 ± 250.8 | 267.5 ± 671.9^a^ |
| Ventilatory Burden 2 (%) | 4.6 ± 11.6 | 3.5 ± 6.2 | 5.1 ± 13.8^a^ |
| PVT Duration (minutes) | 13.7 ± 5.7 | 4.9 ± 1.3 | 16.4 ± 0.8^a^ |
| PVT Mean Response Speed (1/s) | 2.72 ± 0.48 | 3.17 ± 0.31 | 2.57 ± 0.44^a^ |
| PVT Slowest 10% Response Speed (1/s) | 1.77 ± 0.62 | 2.39 ± 0.43 | 1.60 ± 0.52^a^ |
| PVT Lapse Probability (%) | 13.6 ± 29.2 | 16.9 ± 28.5 | 12.6 ± 28.9^a^ |
| PVT Median Response Time (ms) | 361.1 ± 96.6 | 312.1 ± 40.6 | 382.1 ± 103.8^a^ |
| PVT Fastest 10% Response Time (ms) | 292.8 ± 55.1 | 267.1 ± 31.9 | 300.8 ± 55.1^a^ |
| PVT Slowest 10% Response Time (ms) | 600.6 ± 421.8 | 432.1 ± 147.2 | 673.4 ± 463.6^a^ |
| Epworth Sleepiness Scale Score | 8.8 ± 6.5 | 7.5 ± 6.2 | 9.3 ± 6.5^a^ |

Data are presented as *n* (%) or mean ± standard deviation for back-transformed variables. Box-Cox transformation parameters were derived using PA participants only or STAGES participants only for individual cohort columns, and PA and STAGES cohorts combined for the combined cohort column.

^a^*p* < 0.05 between STAGES and PA cohorts using Welch’s t-test.

^b^*p* < 0.05 between STAGES and PA cohorts using the Chi-squared test.

^c^Untransformed hypoxic burden median [interquartile range] was 21.9 [52.8] in the combined cohort, 11.8 [26.0] in the STAGES cohort, and 26.4 [62.8] in the PA cohort.

## Table S2

**Table S2.** Box-Cox transformation values of continuous variables in the combined, STAGES, and Princess Alexandra (PA) Hospital cohorts.

|  | | Combined  Cohort | STAGES  Cohort | PA  Cohort |
| --- | --- | --- | --- | --- |
| Age (years) | $\text{λ}_{\text{1}}$ | 1.16430 | 0.43172 | 1.34379 |
|  | $\text{λ}_{\text{2}}$ | 0 | 0 | 0 |
| Body Mass Index (kg/m^2^) | $\text{λ}_{\text{1}}$ | -0.33036 | -0.98244 | -0.29169 |
|  | $\text{λ}_{\text{2}}$ | 0 | 0 | 0 |
| Total Sleep Time (hours) | $\text{λ}_{\text{1}}$ | 0.60954 | 1.11396 | 0.77524 |
|  | $\text{λ}_{\text{2}}$ | 0 | 0 | 0 |
| Non-Rapid Eye Movement Sleep (%) | $\text{λ}_{\text{1}}$ | 1.18992 | 2.03107 | 1.18992 |
|  | $\text{λ}_{\text{2}}$ | 0 | 0 | 0 |
| Apnea-Hypopnea Index (events/hour) | $\text{λ}_{\text{1}}$ | 0.24459 | 0.26909 | 0.25024 |
|  | $\text{λ}_{\text{2}}$ | 0.00146 | 0.00115 | 0.00146 |
| Airflow Signal-to-Noise Ratio (dB) | $\text{λ}_{\text{1}}$ | -3.96600 | 1.51151 | -3.96600 |
|  | $\text{λ}_{\text{2}}$ | 86.65822 | 0 | 86.65822 |
| Flow Limitation Severity (%) | $\text{λ}_{\text{1}}$ | -3.88166 | 0.31829 | -4.71051 |
|  | $\text{λ}_{\text{2}}$ | 184.59336 | 0 | 229.38792 |
| Hypoxic Burden (%min/hour) | $\text{λ}_{\text{1}}$ | 0.11960 | 0.19294 | 0.12415 |
|  | $\text{λ}_{\text{2}}$ | 0.01192 | 0.00229 | 0.01192 |
| Ventilatory Burden 1 (%eupnea*min/hour) | $\text{λ}_{\text{1}}$ | 0.17221 | 0.19634 | 0.18478 |
|  | $\text{λ}_{\text{2}}$ | 0.04598 | 0.02183 | 0.04598 |
| Ventilatory Burden 2 (%) | $\text{λ}_{\text{1}}$ | -0.06362 | -0.13234 | -0.01701 |
|  | $\text{λ}_{\text{2}}$ | 0.08493 | 0 | 0.05355 |
| PVT Duration (minutes) | $\text{λ}_{\text{1}}$ | -3.90198 | -2.14968 | -5.76346 |
|  | $\text{λ}_{\text{2}}$ | 305.08071 | 0 | 0 |
| PVT Mean Response Speed (1/s) | $\text{λ}_{\text{1}}$ | 1.96056 | 2.78110 | 2.06227 |
|  | $\text{λ}_{\text{2}}$ | 0 | 0 | 0 |
| PVT Slowest 10% Response Speed (1/s) | $\text{λ}_{\text{1}}$ | 1.12450 | 5.60742 | 1.25972 |
|  | $\text{λ}_{\text{2}}$ | 0 | 6.61655 | 0 |
| PVT Lapse Probability (%) | $\text{λ}_{\text{1}}$ | 0.29494 | 0.36772 | 0.26836 |
|  | $\text{λ}_{\text{2}}$ | 0.00100 | 0.00100 | 0.00100 |
| PVT Median Response Time (ms) | $\text{λ}_{\text{1}}$ | -2.03435 | -2.94266 | -2.01130 |
|  | $\text{λ}_{\text{2}}$ | 0 | 0.00030 | 0 |
| PVT Fastest 10% Response Time (ms) | $\text{λ}_{\text{1}}$ | -4.19392 | -3.02315 | -2.75489 |
|  | $\text{λ}_{\text{2}}$ | 288.71759 | 1262.25854 | 0 |
| PVT Slowest 10% Response Time (ms) | $\text{λ}_{\text{1}}$ | -1.01230 | -1.79014 | -1.09681 |
|  | $\text{λ}_{\text{2}}$ | 0 | 0 | 0 |
| Epworth Sleepiness Scale Score | $\text{λ}_{\text{1}}$ | 0.58569 | 0.55351 | 0.60825 |
|  | $\text{λ}_{\text{2}}$ | 0.00024 | 0.00023 | 0.00024 |

The two-parameter Box-Cox transformation is $\text{Y' = }{\text{(Y+}\text{λ}_{\text{2}}\text{)}}^{\text{λ}_{\text{1}}}\text{-1) ÷ }\text{λ}_{\text{1}}$ or $\text{Y' =}\text{ }\text{log}_{\text{e}} \text{(Y+}\text{λ}_{\text{2}}\text{)}$ if $\text{λ}_{1}\text{ = 0}$. For each continuous variable, $\text{λ}_{\text{1}}$ is presented on the first row and $\text{λ}_{\text{2}}$ is presented on the second row.

## Table S3

**Table S3.** Associations between flow limitation severity and psychomotor vigilance task and Epworth Sleepiness Scale outcome variables in the STAGES and Princess Alexandra (PA) Hospital cohorts.

|  | | STAGES  Cohort | PA  Cohort | Percent Difference in Δ |
| --- | --- | --- | --- | --- |
| Mean Response Speed (1/s) | *p* | 0.466 | **0.045** | 59.6% |
|  | Δ | -0.020 | -0.037 |  |
|  | CI | -0.074 to 0.034 | -0.073 to -0.001 |  |
| Slowest 10% Response Speed (1/s) | *p* | 0.177 | **0.008** | 32.1% |
|  | Δ | -0.047 | -0.065 |  |
|  | CI | -0.114 to 0.021 | -0.113 to -0.017 |  |
| Lapse Probability (%) | *p* | 0.604 | **0.011** | 106.6% |
|  | Δ | 0.538 | 1.765 |  |
|  | CI | -1.544 to 2.737 | 0.399 to 3.215 |  |
| Median Response Time (ms) | *p* | 0.565 | 0.074 | 77.6% |
|  | Δ | 1.855 | 4.205 |  |
|  | CI | -4.541 to 8.436 | -0.401 to 8.993 |  |
| Fastest 10% Response Time (ms) | *p* | 0.423 | 0.282 | 9.4% |
|  | Δ | 1.692 | 1.859 |  |
|  | CI | -2.462 to 5.945 | -1.528 to 5.319 |  |
| Slowest 10% Response Time (ms) | *p* | 0.204 | **0.016** | 8.3% |
|  | Δ | 19.585 | 21.289 |  |
|  | CI | -10.516 to 52.457 | 3.847 to 39.841 |  |
| Epworth Sleepiness Scale Score | *p* | 0.388 | **0.021** | 44.0% |
|  | Δ | 0.307 | 0.480 |  |
|  | CI | -0.393 to 1.023 | 0.073 to 0.895 |  |

Analyses used *n* = 324 participants from the STAGES cohort and *n* = 998 participants from the PA cohort. Linear regression models were developed using flow limitation severity, age, sex, body mass index, total sleep time, smoking status, and depression. Statistically significant associations (*p* < 0.05) are in boldface. Percent difference in Δ between the two cohorts was calculated using the equation: ||Δ1| − |Δ2|| ÷ ((|Δ1| + |Δ2|) ÷ 2) × 100. Box-Cox transformation values derived in the combined cohort were used to back-transform outcome variables into comparable units. *p* = *p*-value; Δ = association between one standard deviation increase in flow limitation severity and change in outcome variable; CI = 95% confidence interval of change.

## Table S4

**Table S4.** Associations between predictor variables and psychomotor vigilance task and Epworth Sleepiness Scale outcome variables in the combined STAGES and Princess Alexandra (PA) Hospital cohort.

|  | | Models without site | Models with flow limitation severity and site | | ΔAICc |
| --- | --- | --- | --- | --- | --- |
|  |  | **Flow limitation severity** | **Flow limitation severity** | **Site** |  |
| Mean Response Speed (1/s) | *p* | **0.003** | **0.034** | **0.009** | -4.8 |
|  | Δ | -0.045 | -0.033 | 0.044 |  |
|  | CI | -0.074 to -0.015 | -0.064 to -0.003 | 0.011 to 0.077 |  |
| Slowest 10% Response Speed (1/s) | *p* | 0.088 | **0.004** | **< 0.001** | -17.8 |
|  | Δ | -0.034 | -0.061 | -0.099 |  |
|  | CI | -0.074 to 0.005 | -0.101 to -0.020 | -0.143 to -0.056 |  |
| Lapse Probability (%) | *p* | **0.002** | **0.018** | **0.034** | -2.5 |
|  | Δ | 1.772 | 1.402 | -1.343 |  |
|  | CI | 0.632 to 2.974 | 0.237 to 2.634 | -2.663 to -0.099 |  |
| Median Response Time (ms) | *p* | **0.005** | 0.066 | **0.002** | -7.3 |
|  | Δ | 5.371 | 3.632 | -6.535 |  |
|  | CI | 1.592 to 9.274 | -0.237 to 7.631 | -10.937 to -2.291 |  |
| Fastest 10% Response Time (ms) | *p* | **< 0.001** | 0.153 | **< 0.001** | -50.6 |
|  | Δ | 4.993 | 2.018 | -11.543 |  |
|  | CI | 2.222 to 7.837 | -0.746 to 4.845 | -14.861 to -8.319 |  |
| Slowest 10% Response Time (ms) | *p* | 0.085 | **0.008** | **< 0.001** | -10.8 |
|  | Δ | 12.876 | 20.987 | 30.612 |  |
|  | CI | -1.734 to 28.242 | 5.433 to 37.401 | 13.428 to 48.836 |  |
| Epworth Sleepiness Scale Score | *p* | **0.010** | **0.018** | 0.707 | 1.9 |
|  | Δ | 0.455 | 0.435 | -0.073 |  |
|  | CI | 0.109 to 0.806 | 0.074 to 0.802 | -0.461 to 0.311 |  |

The combined cohort (*n* = 1322) was composed of *n* = 324 participants from the STAGES cohort and *n* = 998 participants from the PA cohort. Linear regression models were developed using flow limitation severity, age, sex, body mass index, total sleep time, smoking status, and depression. “Site” was added as a binary categorical term to compensate for differences between cohort characteristics not captured by standard covariates. PA was the reference site. Statistically significant associations (*p* < 0.05) are in boldface. Box-Cox transformation values derived in the combined cohort were used to back-transform outcome variables into comparable units. *p* = *p*-value; Δ = association between either one standard deviation increase in flow limitation severity or change in site from PA to STAGES, and change in outcome variable; CI = 95% confidence interval of change; ΔAICc = difference in corrected Akaike information criterion between the two models (a negative value indicates the model with site had a better fit than the model without site).

## Table S5

**Table S5.** Association between one standard deviation increase in predictor variables and decreased psychomotor vigilance task (PVT) mean response speed in the combined STAGES and Princess Alexandra (PA) Hospital cohort.

| Model |  | ESS | Sex | Age | Flow Limitation Severity |
| --- | --- | --- | --- | --- | --- |
| Mean Response Speed = 1 + ESS | *p* | **< 0.001** | - | - | - |
|  | Δ | -0.076 | - | - | - |
|  | CI | -0.103 to -0.049 | - | - | - |
| Mean Response Speed = 1 + Sex | *p* | - | **< 0.001** | - | - |
|  | Δ | - | 0.133 | - | - |
|  | CI | - | 0.107 to 0.159 | - | - |
| Mean Response Speed = 1 + Age | *p* | - | - | **0.006** | - |
|  | Δ | - | - | -0.039 | - |
|  | CI | - | - | -0.066 to -0.011 | - |
| Mean Response Speed = 1 + ESS  + Sex + Age + Flow Limitation Severity | *p* | **< 0.001** | **< 0.001** | **0.003** | **0.003** |
|  | Δ | -0.070 | 0.139 | -0.041 | -0.042 |
|  | CI | -0.096 to -0.043 | 0.113 to 0.165 | -0.068 to -0.014 | -0.070 to -0.015 |

The combined cohort (*n* = 1322) was composed of *n* = 324 participants from the STAGES cohort and *n* = 998 participants from the PA cohort. Linear regression models were developed using ESS, sex, age, and flow limitation severity. Statistically significant associations (*p* < 0.05) are in boldface. Box-Cox transformation values derived in the combined cohort were used to back-transform outcome variables into comparable units. In bivariate analyses, ESS, sex, and age were all significantly associated with mean response speed (*p* ≤ 0.006). These results are consistent with previously published literature. Higher ESS scores are associated with decreased PVT performance [16]. It has also been shown that females have slower PVT response speeds than males [10,17]. It has been reported that younger healthy men have faster baseline PVT response speeds than older healthy men [18]. Also, older adults have worse vigilance than younger adults in alternative measures of vigilance [19-21]. In multivariable analysis, all predictor variables were significant (*p* ≤ 0.003). This suggests that flow limitation is significantly associated with PVT performance independent of ESS, sex, and age. *p* = *p*-value; Δ = association between either one standard deviation increase in predictor variable and change in mean response speed; CI = 95% confidence interval of change; ESS = Epworth Sleepiness Scale score.

# Supplementary References

1. Gur RC, Richard J, Hughett P, et al. A cognitive neuroscience-based computerized battery for efficient measurement of individual differences: Standardization and initial construct validation. Journal of Neuroscience Methods. 2010; 187 (2): 254-262.

2. STAGES Cohort Investigator Group. Manual of Procedures (MOP) for STAGES – Stanford Technology, Analytics, and Genomics in Sleep. 2018; Version 2 008/09/2018. <https://sleepdata.org/datasets/stages/files/documentation/STAGES%20MOP%202018-08-09.pdf>.

3. STAGES Cohort Investigator Group. Data Elements for Stanford Technology Analytics and Genomics in Sleep (STAGES) Study. 2017; 1/31/2017. <https://sleepdata.org/datasets/stages/files/m/browser/original/Project%20Descriptions/Data%20Elements%20for%20STAGES%202017-06-12.docx>.

4. Basner M, Dinges DF. Maximizing sensitivity of the psychomotor vigilance test (PVT) to sleep loss. Sleep. 2011; 34 (5): 581-591.

5. Staykov E, Mann DL, Duce B, et al. Increased flow limitation during sleep is associated with increased psychomotor vigilance task lapses in individuals with suspected obstructive sleep apnea. Chest. 2023.

6. Basner M, Mollicone D, Dinges DF. Validity and sensitivity of a brief psychomotor vigilance test (PVT-B) to total and partial sleep deprivation. Acta Astronautica. 2011; 69 (11): 949-959.

7. Mann DL, Terrill PI, Azarbarzin A, et al. Quantifying the magnitude of pharyngeal obstruction during sleep using airflow shape. Eur Respir J. 2019; 54 (1): 1802262.

8. Staykov E, Mann DL, Kainulainen S, et al. Nasal Pressure Derived Airflow Limitation and Ventilation Measurements are Resilient to Reduced Signal Quality. Annu Int Conf IEEE Eng Med Biol Soc. 2023.

9. Azarbarzin A, Sands SA, Stone KL, et al. The hypoxic burden of sleep apnoea predicts cardiovascular disease-related mortality: the Osteoporotic Fractures in Men Study and the Sleep Heart Health Study. Eur Heart J. 2019; 40 (14): 1149-1157.

10. Kainulainen S, Duce B, Korkalainen H, et al. Severe desaturations increase psychomotor vigilance task-based median reaction time and number of lapses in obstructive sleep apnoea patients. Eur Respir J. 2020; 55 (4): 1901849.

11. Labarca G, Vena D, Hu W-H, et al. Sleep Apnea Physiological Burdens and Cardiovascular Morbidity and Mortality. Am J Respir Crit Care Med. 2023; 208 (7): 802-813.

12. Parekh A, Kam K, Wickramaratne S, et al. Ventilatory Burden as a Measure of Obstructive Sleep Apnea Severity Is Predictive of Cardiovascular and All-Cause Mortality. Am J Respir Crit Care Med. 2023; 208 (11): 1216-1226.

13. Box GEP, Cox DR. An Analysis of Transformations. Journal of the Royal Statistical Society Series B (Methodological). 1964; 26 (2): 211-252.

14. Ribeiro Jr PJ, Diggle PJ. *Analysis of Geostatistical Data.* The geoR package; 2022.

15. R Core Team R. *R: A language and environment for statistical computing.* R Foundation for Statistical Computing, Vienna, Austria; 2022.

16. Shattuck NL, Matsangas P. Psychomotor vigilance performance predicted by Epworth Sleepiness Scale scores in an operational setting with the United States Navy. Journal of Sleep Research. 2015; 24 (2): 174-180.

17. Blatter K, Graw P, Münch M, Knoblauch V, Wirz-Justice A, Cajochen C. Gender and age differences in psychomotor vigilance performance under differential sleep pressure conditions. Behavioural brain research. 2006; 168 (2): 312-317.

18. Adam M, Rétey JV, Khatami R, Landolt H-P. Age-Related Changes in the Time Course of Vigilant Attention During 40 Hours Without Sleep in Men. Sleep. 2006; 29 (1): 55-57.

19. Parasuraman R, Giambra L. Skill development in vigilance: Effects of event rate and age. Psychology and Aging. 1991; 6 (2): 155-169.

20. Parasuraman R, Nestor P, Greenwood P. Sustained-attention capacity in young and older adults. Psychology and Aging. 1989; 4 (3): 339-345.

21. Surwillo WW, Quilter R. Vigilance, age, and response-time. The American Journal of Psychology. 1964; 77 (4): 614-620.
